# Micro-shear bond strength of different calcium silicate materials to bulk-fill composite

**DOI:** 10.7717/peerj.15183

**Published:** 2023-03-29

**Authors:** Seda Falakaloğlu, Merve Yeniçeri Özata, Gianluca Plotino

**Affiliations:** 1 Department of Endodontics, Faculty of Dentistry, Istanbul Health and Technology University, Istanbul, Turkey; 2 Current Affiliation: Afyonkarahisar Health Sciences University, Faculty of Dentistry, Department of Endodontics, Afyonkarahisar, Turkey; 3 Department of Endodontics, Faculty of Dentistry, Dicle University, Diyarbakır, Turkey; 4 Private Practice, Grande Plotino & Torsello—Studio di Odontoiatria, Via Calabria, Rome, Italy

**Keywords:** Biodentine, Bio MTA+, Bulk-fill composite, TheraCal PT, TheraCal LC

## Abstract

**Introduction:**

This study aimed to compare the micro-shear bond strength (µSBS) performances of two resin-based calcium silicate-based cement (CSC) (TheraCal PT and TheraCal LC), Biodentine, and two modified-MTA CSC materials (NeoMTA 2 and BioMTA+) to bulk-fill restorative material.

**Materials and Methods:**

Fifty 3D printed cylindrical resin blocks with a central hole were used (2 mm in depth and 4 mm in diameter). CSCs were placed in the holes (per each group *n* = 10) and incubated for 24 h. Cylindrical polyethylene molds (2 mm in height and diameter) were used to place the bulk-fill restorative materials on the CSCs and polymerize for 20 s. Then, all specimens were incubated for 24 h at 37 °C at a humidity of 100%. Specimen’s µSBSs were determined with a universal testing machine. Data were analyzed with one-way ANOVA (Welch) and Tamhane test.

**Results:**

Statistically higher µSBS was found for TheraCal PT (29.91 ± 6.13 MPa) (*p* < 0.05) respect to all the other materials tested. TheraCal LC (20.23 ± 6.32 MPa) (*p* > 0.05) reported higher µSBS than NeoMTA 2 (11.49 ± 5.78 MPa) and BioMTA+ (6.45 ± 1.89 MPa) (*p* < 0.05). There was no statistical difference among TheraCal LC, NeoMTA 2 and Biodentine (15.23 ± 7.37 MPa) and between NeoMTA 2 and BioMTA+ (*p* > 0.05).

**Conclusion:**

Choosing TheraCal PT as the pulp capping material may increase the adhesion and µSBS to the bulk-fill composite superstructure and sealing ability.

## Introduction

Operative interventions to the pulp organ, microleakage and toxicity of restorative materials may negatively affect the dentin pulp complex. The procedure which aims to preserve pulp vitality and involves the placement of specific material is called pulp capping (PC) (Briso et al., 2006). Calcium hydroxide (Ca(OH)_2_) was reported as the gold standard in PC applications, until the introduction of new materials with better biological and physical properties. The disadvantages of this material are insufficient adhesion to the dentine, the porosity of the hydroxyapatite bridges it forms (Liu et al., 2021), insufficient sealing (Hilton, 2009), high solubility, and uncontrolled necrotic zone formation as a result of its local effect on the pulp tissue (Hargreaves, Goodis & Tay, 2012). Calcium silicate-based cements (CSC), which eliminate many of these negative features, have started to be included in standard PC procedures (Beegum et al., 2021; Manaspon et al., 2021; Nowicka et al., 2013). Tricalcium silicate cements (t-CSC) form a homogeneous dentin bridge in the pulp tissue without an inflammatory response (Nowicka et al., 2013; Tran et al., 2012).

Biodentine (Septodont, Saint-Maur-des-Fossés, Creteil, France) is a fast setting CSC that can replace dentin tissue. Contrary to the long setting time of mineral trioxide aggregate (MTA), Biodentine has a wide usage area in endodontics as it hardens in a short time like 12 min and releases calcium ions (Dawood et al., 2017). TheraCal LC (Bisco Inc., Shaumburg, IL, USA) release calcium ions. In addition, when this material is applied to the pulp cell, the changes in protein expression, metabolism, and morphology of the cell can be tolerated by this pulp cell (Beegum et al., 2021). Today, despite all these features, the use of TheraCal LC is controversial for PC because of cytotoxicity to pulp tissue (Jeanneau et al., 2017; Manaspon et al., 2021; Rodríguez-Lozano et al., 2021; Sahin, Saygili & Akcay, 2021).

The new chemical formulation of resin-based TheraCal PT (Bisco Inc., Schaumburg, IL, USA) facilitates calcium release thanks to its hydrophilic matrix structure. The hydrophilic matrix consists of calcium silicate with the same structure as Portland cement (Bisco, 2021). It has been reported that this matrix structure facilitates calcium release by providing dentin bridge formation (Okabe et al., 2006). The application with automix cannula tips, dual-cure polymerization, the maximum setting time of 5 min and the ability to apply permanent restorative material in a single session represents some advantages of this material (Bisco, 2021). The manufacturer recommends using the product as a liner in indirect/direct PC and pulpotomy procedures. In an *in vitro* study, the cytocompatibility and bioactive properties of TheraCal PT were compared with other CSCs. Unlike TheraCal LC, TheraCal PT showed similar properties to Angelus MTA and Biodentine on human dental pulp stem cells (Rodríguez-Lozano et al., 2021; Sanz et al., 2021).

MTA-alternative CSC materials with radiopacifiers or nanoparticles are available clinically. Bio MTA+ (Cerkamed, Stalowa Wola, Poland) is a modified MTA material containing hydroxyapatite components and nanoceramic particles with a reduced grain size three times (Dawood et al., 2017). According to the manufacturer, the content of silicon, hydroxyapatite and calcium compounds promote dentine regeneration (Cerkamed, 2021). In addition, more mineral phase and apatite layer are formed during its hardening than TheraCal LC (Peskersoy, Lukarcanin & Turkun, 2021). The tooth discoloration problem is solved by the use of tantalite instead of bismuth oxide as a radioactive pacifier in NeoMTA 2 (NuSmile Avalon Biomed, Bradenton, FL, USA). This material triggers the healing process by stimulating hydroxyapatite in dentin (NuSmile, 2020).

In the pulp capping procedure, providing sterile conditions and a sealed superstructure is an important step for dentin bridge formation and pulp repair (Oskoee et al., 2014). Otherwise, the presence of bacteria and insufficient adhesion between the restorative material and the CSC endanger the vitality of the pulp. Therefore, the bond strength between these materials is important (Ajami et al., 2013; Oskoee et al., 2014). Moreover, the bond strength of the CSCs to restorative material can be affected by surface treatments, replacement of adhesive systems, and the contents of CSCs (Deepa et al., 2016; Hilton, 2009; Peskersoy, Lukarcanin & Turkun, 2021).

Composite resins are placed in deep cavities in thin layers, and each layer is polymerized separately. However, the layering technique may lead to failure of the PC procedure due to the risk of interlayer failure, contamination, and interlayer gap formation (Abbas et al., 2003; Sarrett, 2005). In bulk-fill resin composites, sufficient polymerization can be achieved in a single layer of 4 or 5 mm (Ilie, Keßler & Durner, 2013). Filtek™ Bulk Fill Posterior Restorative (3M, St. Paul, MN, USA) material has a high viscosity and less shrinkage stress. Also, when used with self-etch, it can significantly reduce the duration of the PC procedure (Sabbagh, McConnell & McConnell, 2017).

The use of materials that can prevent leakage by better bonding to the superstructure is essential for PC. The physical properties of TheraCal PT, whose bioactivity and cytotoxicity have been studied and promising, will affect the bond strength and thus leakage. To our best knowledge, there is no micro-shear bond strength (µSBS) study in the literature comparing this material with other CSCs. Moreover, the joint of bulk-fill restorative material with powder-liquid and light or dual-curing PC materials has yet to be fully discovered. Therefore, this study aimed to compare *in vitro* µSBS of two resin-based modified CSCs (TheraCal PT and TheraCal LC) and three CSCs (Biodentine, NeoMTA 2, and BioMTA+) to a bulk-fill composite and to evaluate their bond failure mode under dental operating microscope. The tested null hypothesis was that no statistically significant differences might be found among the five tested CSCs regarding µSBS value.

## Materials and Methods

The sample size calculation was performed with G Power 3.1 software (Heinrich Heine University, Dusseldorf, Germany) (α = 0.05, 1 – β = 0.95, effect size of 0.752). The calculation indicated that the sample size for each group must be a minimum of eight resin blocks (Palma et al., 2021). The materials used in this study are listed in Table 1.

**Table 1:** General information according to material brands used in the present study.

Material	LOT number	Manufacturer location	Type	Composition	Application steps
TheraCal PT	2100003403	Bisco, Inc. 1100 W. Irving Park Rd. Schaumburg, IL 60193, USA	Single paste dual-cured, resin modified CSC	Silicate glass-mix cement (50–75 wt%) (Unknown CAS no), Polyethylene Glycol Dimethacrylate (10–30 wt%) (CAS no. 25852-47-5) Bis-GMA (5–10 wt%) (CAS no.1565-94-2), Barium zirconate (1–5 wt%) (CAS no. 12009-21-1), Ytterbium fluoride (CAS no. 13760-80-0), Initiator.	Inject the material into the cavity in 1 mm increments Light cure each increment for 20 s.
TheraCal LC	2100001487	Bisco, Inc. 1100 W. Irving Park Rd. Schaumburg, IL 60193, USA	Single paste light-cured, resin modified CSC	Portland cement (30–50 wt%) (CAS no. 65997-15-1), Strontium glass, Fumed silica, Barium sulphate, Barium zirconate (1–5 wt%) (CAS no. 12009-21-1), Bis-GMA (5–10 wt%) (CAS no. 1565-94-2), PEGDMA (CAS no. 25852-47-5).	Inject the material into the cavity in 1 mm increments Light cure each increment for 20 s.
Biodentine	B28023	Septodont. 58 rue du Pont de Creteil 94100 Saint Maur des Fosses, France	CSC	**Powder:** Calcium carbonate (50–100 wt%) (CAS no. 471-34-1), Calcium oxide (<0.5 wt%) (CAS no. 1305-78-8), Zirconium dioxide (5–10 wt%) (CAS no. 1314-23-4), Oxide filler, Iron oxide shade. **Liquid:** Aqueous solution of a hydrosoluble polymer with calcium chloride (CAS no. 0035-04-8).	Pour five drops liquid from into the capsule. Place the capsule on a mixing device and mix 30 s.
NeoMTA 2	2022051801	NuSmile Ltd. 3315 W 12th Street, Houston, TX 77008 USA	CSC	**Powder:** Tricalcium silicate (<50 wt%) (CAS no. 12168-85-3), Dicalcium silicate (<20 wt%) (CAS no.10034-77-2), Tantalite (<50 wt%) (CAS no. 1314-61-0), Calcium sulfate (<5 wt%) (CAS no. 7778-18-9), Tricalcium aluminate (<5 wt%) (CAS no. 12042-78-3). **Liquid:** Water and proprietary polymers.	Mix powder/liquid ratio in 2:1 Apply a layer of at least 1.5 mm thick.
BioMTA+	2811221	Cerkamed. Wojciech Pawłowski 37-450 Stalowa Wola, Kwiatkowskiego, Poland	Hydroxyapatite and nanoparticle structure* Modified CSC^†^	**Powder:** Calcium oxide (<50 wt%) (CAS no. 1305-78-8), Hydroxyapatite (35–40 wt%), Oxides of (<10 wt%): silicon, iron, aluminum, sodium, potassium, bismuth, magnesium, zirconium; calcium phosphate. **Liquid:** Purified water, calcium catalyst.	Mix the powder with one to two drops of the liquid for 30 s until it reaches a consistency of soft plasticine.
Bond Force II	161	Tokuyama Dental Corp. 38-9,Taitou 1-chome, Taitou-ku, Tokyo, 110-0016, Japan	Adhesive system	Self-reinforcing 10-MDP (CAS no. 85590-00-7), Bis-GMA (CAS no. 1565-94-2), TEGDMA (CAS no. 109-16-0), HEMA (CAS no. 868-77-9), alcohol, water	Ensure the adhesive covers all surfaces where the bulk-fill resin will be applied. Air dry for 10 s. Light cure for 10 s or more, keeping the light tip within a distance of 2 mm from the surface.
Filtek Bulk-fill posterior	NA85802	3M. Oral Care Solutions Division, 3M Center, St. Paul, MN 55144-1000, USA	Resin composite	**Filler:** Zirconia/silica (unknown), ytterbium trifluoride (CAS no. 13760-80-0) **Resin matrix:** AUDMA (CAS no. 1431303-59-1), AFM (CAS no. 1429648-13-4), DDDMA (CAS no. 72829-09-5), UDMA (CAS no. 72869-86-4)	Placement the bulk fill up to 5 mm deep. Light cure for 20 s for Class I cavity.

**Notes:**

Bis-GMA, bisphenol A-glycidyl dimethacrylate (C_29_H_36_O_8_); PEGDMA, polyethylene glycol dimethacrylate (C_3_H_5_C(O)(OCH_2_CH_2_)_n_OC(O)C_3_H_5)_; 10-MDP, 10-methacryloyloxydecyl dihydrogen phosphate (C_14_H_27_O_6_P); TEGDMA, Triethylene glycol dimethacrylate (CH_2_=C(CH_3_)COO(CH_2_CH_2_O)_3_COC(CH_3_)=CH_2_); HEMA, 2-hydroxyethyl methacrylate (CH_2_=C(CH_3_)COOCH_2_CH_2_OH); AUDMA, aromatic urethane dimethacrylate; AFM, addition fragmentation monomers; DDDMA, 1, 12-Dodecanediol dimethacrylate (C_20_H_34_O_4_); UDMA, urethane dimethacrylate (C_23_H_38_N_2_O_8_); CSC, calcium silicate cement; CAS, Chemical abstracts service.

*Manufacturer (Cerkamed, 2021).

^†^Peskersoy, Lukarcanin & Turkun (2021).

For the µSBS test, fifty 3D-printed cylindrical resin blocks of 35 mm in height and 25 mm in diameter with a hole into the center of 4 millimeters (mm) diameter and 2 mm depth were prepared. The blocks were divided into five groups (*n* = 10) and CSCs were placed in the holes of the blocks using a dental operative microscope (OMS 2380; Zumax, Suzhou, China) under 10× magnification. The excess materials were removed from the surface. Automixing syringe CSC (TheraCal PT) and single-paste CSC (TheraCal LC) were applied in two layers of 1 mm and each layer was polymerized with curing light (DTE Lux E-Guilin Woodpecker Medical Instrument Co. Ltd., Guilin China) with a spectral range of 420–480 nm and an irradiance of 1,200 mW/cm^2^ for 20 s. Powder-liquid CSCs (Biodentine, NeoMTA 2 and BioMTA+) were mixed according to the manufacturer’s instructions (Table 1). Then, each bioceramic sample was allowed to be set in a humidor at 37 °C and 100% humidity for 24 h.

A self-etch adhesive (Bond Force II; Tokuyama Dental, Tokyo, Japan) was applied to the surface of the CSCs with active brushing for 10 s and was gently air-dried over the entire surface. Then it was polymerized using a light-emitting diode curing unit for 10 s. Then, the bulk-fill posterior composite (Filtek, 3M ESPE, St. Paul, MN, USA) was placed in 2 mm × 2 mm sized cylindrical polyethylene molds (according to the ISO 4049) positioned on the surface of the materials following the manufacturer’s recommendations and polymerized for 20 s. Finally, all the samples were stored at 37 °C with 100% humidity for 24 h, before proceeding to the µSBS test.

Specimens were fixed to the universal testing device (Instron; Shimadzu Corp., Chiyoda-Ku, Tokyo, Japan), and µshear mode was selected. Then, a compression load was applied parallel to the adhesive interface at a 1 mm/min crosshead speed until the failure occurred and the value of each sample was measured in Newton. Then, the µSBS value for each sample was calculated as the force divided by the surface area and recorded in MPa.

**Figure 1:**
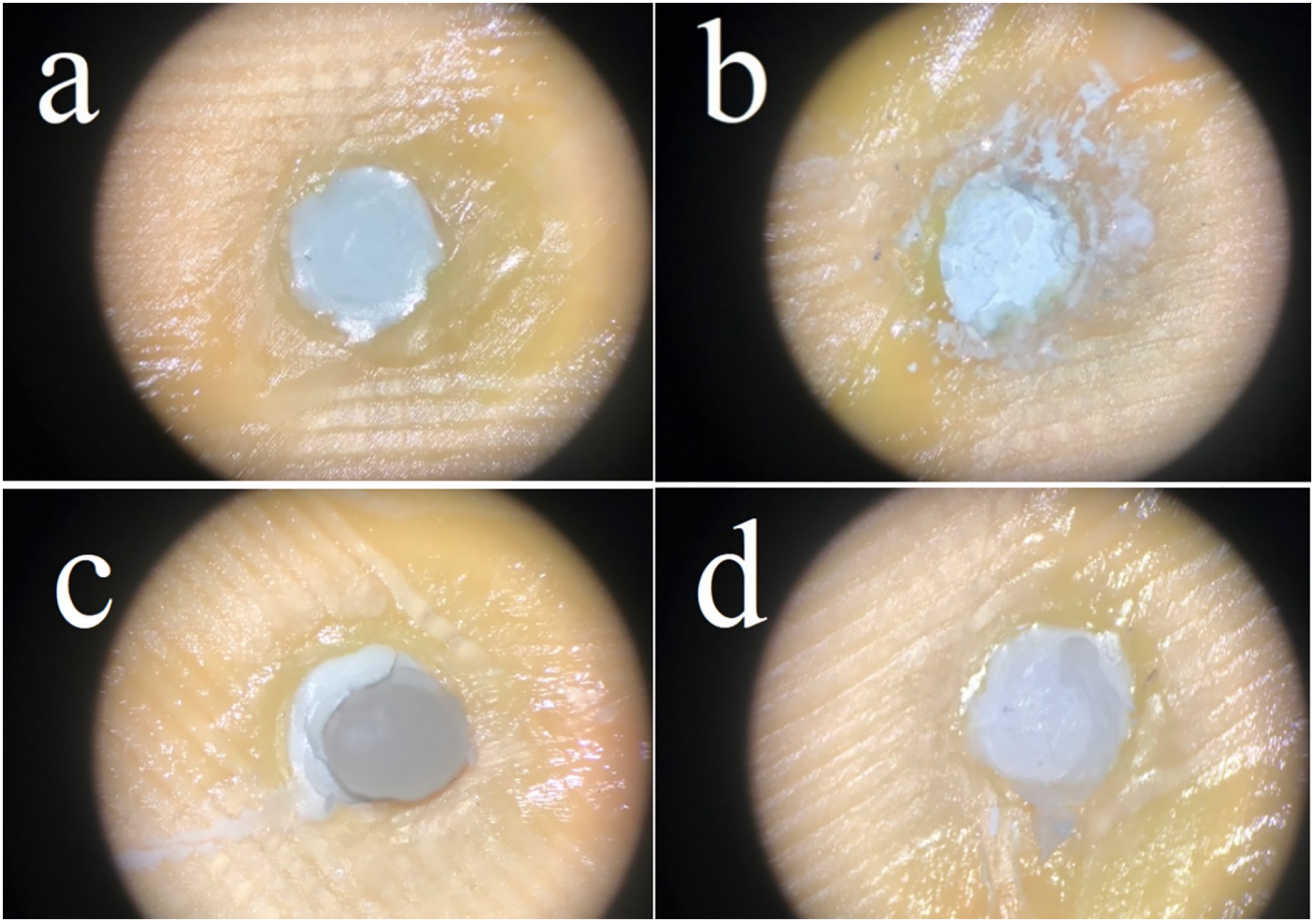
Failure types; adhesive failure (A), cohesive failure in CSC material (B), cohesive failure in bulk-fill material (C), mixed failure (D).

The sample fracture surfaces were evaluated by a single operator using a dental operative microscope (OMS 2380; Zumax, Suzhou, China) under ×19.8 magnification and defined according to their failure types as follows:

a) Adhesive failure: Fracture between CSC and bulk-fill material with no resin remnants,
b) Cohesive failure in CSC material,
c) Cohesive failure in bulk-fill material,
d) Mixed failure: Combination of cohesive and adhesive failure (Fig. 1).

### Statistical analysis

The data showed normal distribution according to the Shapiro-Wilk test. Since the variances were not homogeneous according to Levene’s test, the data were compared with the one-way ANOVA (Welch) test. Multiple comparisons were made using the Tamhane test. Analysis were done using SPSS 21.0 Software (IBM Corp, Armonk, NY, USA). The alpha-type error was set at 5%.

## Results

**Figure 2:**
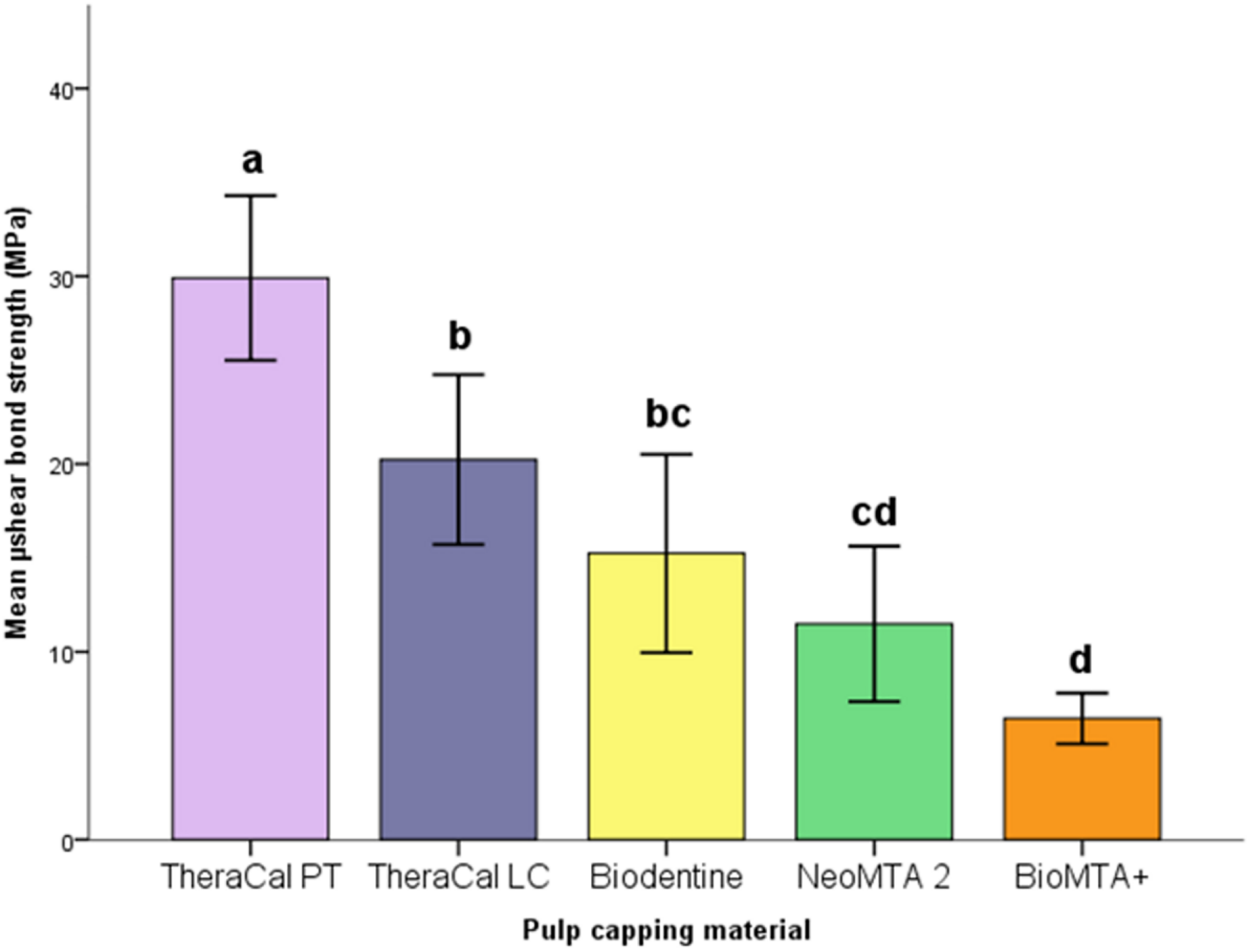
Box plot representation of mean and confidence interval (CI) SBS values. Error bars: 95% CI. The values with same letters were not significantly different at *p* < 0.05.

The descriptive statistics in each group were listed in Table 2 and shown in Fig. 2. µSBS of TheraCal PT was statistically higher than other groups (*p* < 0.05). There was no significant difference between TheraCal LC and Biodentine (*p* = 0.727) being both higher than BioMTA+ (*p* < 0.05). TheraCal LC had higher µSBS than NeoMTA 2 (*p* = 0.046) and BioMTA+ (*p* < 0.001). No statistically significant difference between Biodentine and NeoMTA 2 (*p* = 0.920) and between NeoMTA 2 and BioMTA+ (*p* = 0.216) was found.

**Table 2:** Mean, standard deviation (SD), minimum and maximum SBS values of the tested pulp capping materials (MPa).

Pulp capping material	*n*	Mean ± SD	Minimum	Maximum
TheraCal PT	10	29.91 ± 6.13^**a**^	21.70	40.18
TheraCal LC	10	20.23 ± 6.32^**b**^	11.71	30.29
Biodentine	10	15.23 ± 7.44^**bc**^	5.77	24.60
NeoMTA 2	10	11.49 ± 5.78^**cd**^	3.64	22.07
BioMTA+	10	6.45 ± 1.89^**d**^	2.84	8.48

**Notes:**

The values with same letters were not significantly different at *p* < 0.05.

The failure type distribution was shown in Table 3. The most common type of failure in the TheraCal PT and Biodentine groups were mixed types. Adhesive failure was registered in only one sample of TheraCal LC group and most of the observed failures for TheraCal LC were cohesive fractures within the restorative material. Most of the observed failure types in NeoMTA 2 and BioMTA+ were cohesive fractures within the bioceramic material.

**Table 3:** Distribution of failure types by pulp capping materials.

Pulp capping material	*n*	Adhesive failure	Cohesive failure in CSC material	Cohesive failure in bulk-fill material	Mixed failure
TheraCal PT	10	–	3	2	5
TheraCal LC	10	1	2	5	2
Biodentine	10	–	1	1	8
NeoMTA 2	10	–	8	2	–
BioMTA+	10	–	9	1	–

## Discussion

The present study tested the µSBS performance of a novel resin-based dual-cure TheraCal PT and its predecessor resin-based light-cure TheraCal LC, the second-generation hydraulic CSC Biodentine, the modified BioMTA+ and NeoMTA 2. In one of the few studies regarding the newly introduced TheraCal PT material, it has been shown that it had bioactive properties to maintain vitality and it was cytocompatible (Rodríguez-Lozano et al., 2021). This material, which has good biological properties, must also have good physical properties to support a leak-proof restorative filling. According to the result of the present study, TheraCal PT (29.91 ± 6.13 MPa) had better µSBS than the other four CSCs. Thus, the null hypothesis was rejected. In SEM-EDS analyze study determined that TheraCal PT contained more zirconium and silicon than TheraCal LC, and that TheraCal PT did not contain fluoride (Rodríguez-Lozano et al., 2021). The reason why TheraCal PT shows better µSBS than TheraCal LC may be due to the differences in its content. According to the information given by the manufacturer, the cement ratio for TheraCal PT is between 50–75% by weight, while for TheraCal LC, it is between 30–50% (Bisco, 2021). High molecular weight may have reduced light transmission in TheraCal PT but may have increased bond strength by preventing residual monomer thanks to its dual-cure nature. Giraud et al. (2018) attributed the pulpal toxic effects of TheraCal LC to non-polymerized resin monomers. Therefore, TheraCal PT can be recommended as a liner in PC procedures in terms of bond strength, but studies are needed on its physicochemical, impermeability, setting, cell viability and ion release properties. PEGDMA, which is present in TheraCal LC but not in TheraCal PT, is a hydrophilic monomer. It can tolerate dentin moisture (Bisco, 2021; Guo et al., 2007). If we had examined the bond strength to dentin, we might have seen different results.

In the present study, TheraCal PT showed 29.91 ± 6.13 MPa and TheraCal LC 20.23 ± 6.32 MPa bond strength. Bond strength values to dentine tissue have been shown to vary between 17–20 MPa for a marginally fit sealed restoration (Davidson, De Gee & Feilzer, 1984). TheraCal LC exhibited higher µSBS than Biodentine (15.23 ± 7.44 MPa), although it was insignificant. Studies were stating that TheraCal LC has superior µSBS properties than Biodentine (Cengiz & Ulusoy, 2016; Deepa et al., 2016; Jain & Tiku, 2019; Singla & Wahi, 2020) and there was a study showing no differences between them (Karadas & Atıcı, 2020). In a study, the authors observed that the bottom layer of TheraCal LC was not sufficiently polymerized, although it was applied in a 1 mm layer (Nilsen et al., 2017). Although the adhesion of TheraCal LC to the superstructure increased due to its resin content, the deficiency of polymerization may have reduced the bond strength. Also, unlike MTA, Biodentine’s liquid contains a mixture of distilled water, calcium chloride (accelerator of the setting reaction) and a water-soluble polymer (a mixture that reduces the amount of water required for the setting reaction) (Kaup et al., 2015). Thanks to the reduced distilled water content, Biodentine may have provided similar bond strength to the resin-containing TheraCal LC by providing more outstanding adhesion to the adhesive material than MTA.

The permanent filling can be performed immediately on TheraCal PT and TheraCal LC materials (Bisco, 2021). Although the setting time is 12 min, Biodentine has been reported to reach final hardness in 14 days (Mehra et al., 2020). Moreover, the setting time was 15 min for NeoMTA 2 and 2 h for BioMTA+ by the manufacturer (Cerkamed, 2021; NuSmile, 2020). However, there has yet to be a study on the time to reach final hardness. Therefore, in the present study, all bioceramic sample were stored for 24 h to allow complete hardening. In the literature, studies examine the bond strength between Biodentine and its superstructure for periods ranging from 24 h to one month and immediately after mixing (Nekoofar et al., 2018). When the µSBS of the filling material applied on Biodentine was measured in 12 min and 24 h, it was observed that the strength increased (Kudva et al., 2022; Nekoofar et al., 2018) or did not change (Odabaş, Bani & Tirali, 2013) in different studies. In our study, we tried to prevent time from being an influential factor in SBS by keeping the incubation time constant.

A study stated that MTA promoted cell viability, cell migration, and cell attachment and induced odonto/osteogenic differentiation of human dental pulp stem cells compared to the control group (Rodríguez-Lozano et al., 2021). Despite its good biological properties, in the present study, NeoMTA 2 did not exhibit superior µSBS values as TheraCal PT and TheraCal LC. This may be because the resin components in TheraCal PT and TheraCal LC provide chemical bonding to the dental resin bulk-fill composite and the resinous components in applied dental adhesives (El-Refai, 2016). On the other hand, it was stated in a study that the adhesive between the resin composite and MTA reduces the bonding efficiency by creating shrinkage stress (Cervino et al., 2017). Moreover, the distilled water in MTA can prevent the dental adhesive, a hydrophobic material, from adhesion to MTA. Considering all these, it is understandable that both MTA brands used in our study have less bond strength than CSC materials containing resin.

In a study comparing the predecessor NeoMTA and Biodentine, Biodentine showed statistically significantly higher values. NeoMTA 2 showed shear bond strength similar to Biodentine in our study. The difference between two MTAs may be due to different content (tantalite opacifier) and physical feature (low water solubility) in later NeoMTA 2 (NuSmile, 2020). In a study examining the bond strength of four different bioceramic materials (ProRoot MTA, NeoMTA 2, TotalFill, and NeoPutty) to two different materials (Filtek Z350 XT and GC Fuji II LC) at two different intervals (immediate and delayed), while NeoMTA 2 showed mean 8.57 MPa bond strength in the immediate application, it showed 12.01 MPa bond strength in the delayed application (Alqahtani et al., 2022). In our study, the mean µSBS value in the NeoMTA 2 group, which was incubated for 24 h, was 11.49 MPa. This value is entirely agreeing with the previous study.

Covaci et al. (2020) compared the SBS of two PC materials to dentin, TheraCal LC (1.27 ± 0.69 MPa) found to be significantly better than BioMTA+ (0.22 ± 0.13 MPa). Our study provided a result supporting this finding for TheraCal LC (20.23 ± 6.32 MPa) and BioMTA+ (6.45 ± 1.89 MPa). The reason for the lower MPa values in this study compared to our study may be that the SBS of the CSC to the orthodontic button was investigated or that no adhesive material was applied between the CSC and the orthodontic button. In a study that compared the bond strength of nanoparticle-contained and micro-hybrid structure resin restorative materials, authors stated that the nanoparticle content significantly reduced the SBS of the material (Junior, Ferracane & Della Bona, 2009). Although there is no data to support this interpretation in the literature for CSCs, BioMTA+, the only CSC containing nanoparticles, may have reduced the µSBS compared to other materials in our study. In addition, considering the maximum and minimum SBS values for pulp capping materials, none of the samples in the BioMTA+ group showed a value in the optimum bond strength range (17–20 MPa) (Davidson, De Gee & Feilzer, 1984). This may increase the risk of leakage between the material and the bulk-fill.

The mixed fracture was seen in 50% of the sample in TheraCal PT, which showed high bond strength. Cohesive failure was observed in the bulk-fill material in 50% of the sample in TheraCal LC, which showed the highest values after TheraCal PT. Mixed failure was seen at 80% in Biodentine. Failure types were cohesive in the CSC material with a rate of 80% in NeoMTA 2 and 90% in BioMTA+. The only material in which all failure types were observed was TheraCal LC. We did not observe a relationship between the results of SBS values and failure types.

One of the limitations of this study was that we did not use real dentine tissue, because the organic and inorganic components of dentin can affect the bond strength. In addition, holes of standard diameter and depth were drilled in the center of the blocks. It has been reported that bulk-filled cavities of different depths drilled into bovine teeth affect restorative material’s SBS (He, Shimada & Tagami, 2007). Thus, the hole design was one of the strengths of this study to ensure standardization, but one of its weaknesses was that this design did not reflect clinical conditions. Another limitation of this study is that shear bond tests did not determine the strength of the materials alone. Further studies supported by orthogonal tests are needed.

## Conclusion

The situation is complex given that the CSC must be bonded to dentin and resin-based material and that this structure must maintain integrity. This study investigated only the bond strength between five different CSCs and bulk-fill material. Under the conditions of this study, TheraCal PT showed statistically higher µSBS to bulk-fill composite than TheraCal LC, Biodentine, NeoMTA 2, and BioMTA+. Considering that the bond strength to dentin for leakproof restoration varies between 17–20 MPa, TheraCal PT shows promise owing to good sealing property. Although the bond strength of TheraCal LC material, which is a resin-based CSC, is expected to be better, it showed similar bond strength with Biodentine, a material that has proven its success in endodontics. Although both MTA materials seem to be less successful than the others due to cohesive failure in CSC and lower µSBS values, they can give good results in terms of bond strength when different surface treatments are applied or when the adhesive material is changed.

## Supplemental Information

10.7717/peerj.15183/supp-1 Supplemental Information 1 Dataset.

10.7717/peerj.15183/supp-2 Supplemental Information 2 Visualization of pulp capping materials used in the study, study’s set-up, and the results.

## Additional Information and Declarations

### Data Availability

The following information was supplied regarding data availability: The raw measurements are available in the Supplemental File.
