# Supplementary figures and images for: Micro-shear bond strength of different calcium silicate materials to bulk-fill composite
Source: PeerJ. 2023 Mar 29;11:e15183. doi: 10.7717/peerj.15183 (PMC10066686; doi:10.7717/peerj.15183)

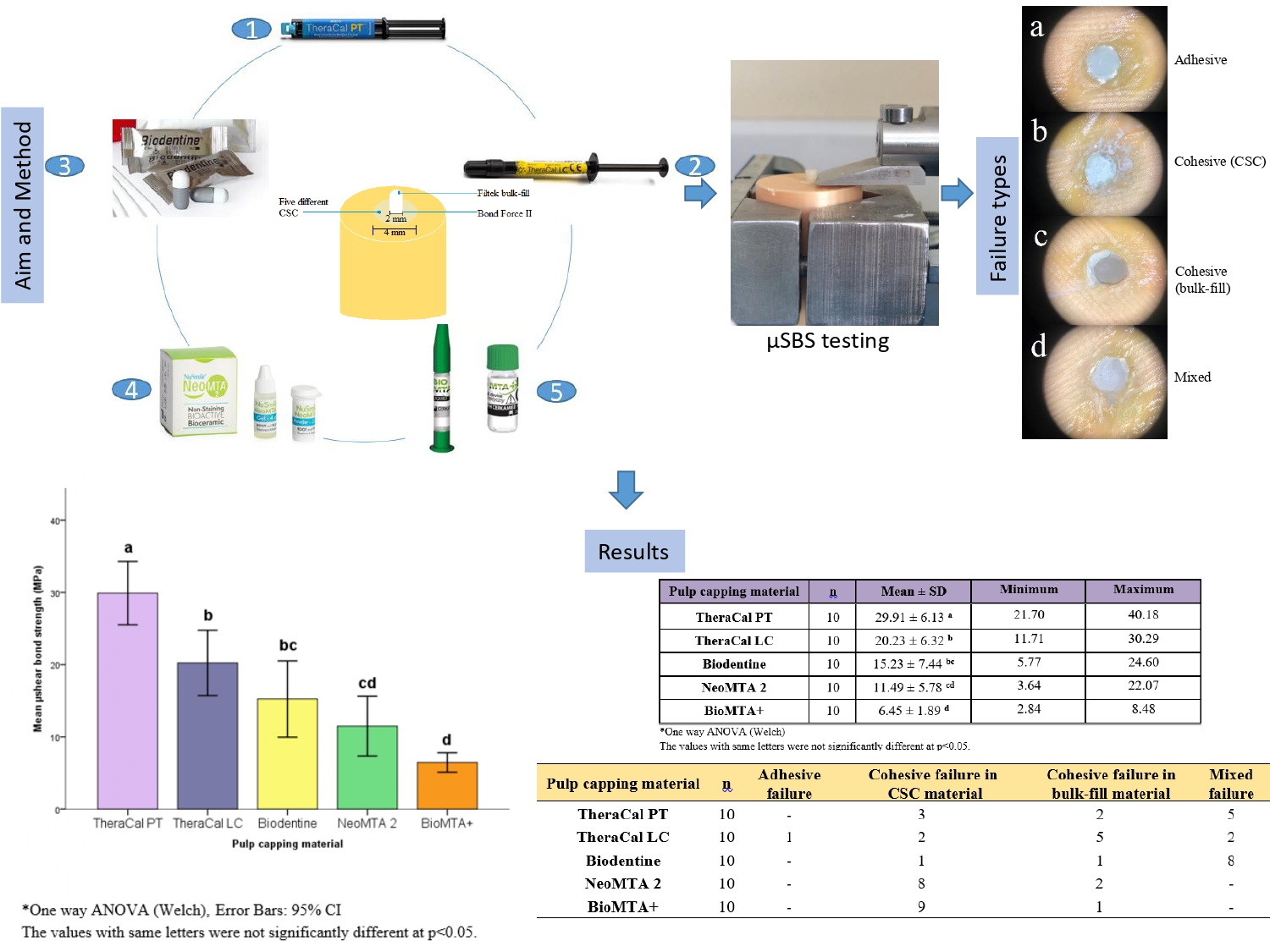

Supplement: Supplemental Information 2 [file peerj-11-15183-s002.jpg]
